# The Upper Range Limit of Alien Plants Is Not in Equilibrium with Climate in the Andes of Central Chile

**DOI:** 10.3390/plants11182345

**Published:** 2022-09-08

**Authors:** Estefany Goncalves, Ileana Herrera, Jake Alexander, Milen Duarte, Lohengrin A. Cavieres, Luis Morales-Salinas, Ramiro O. Bustamante

**Affiliations:** 1 Laboratorio de Ecología Geográfica, Facultad de Ciencias, Universidad de Chile, Santiago 7800003, Chile; 2 Instituto de Ecología y Biodiversidad (IEB), Barrio Universitario, Concepción 7800003, Chile; 3 Escuela de Ciencias Ambientales, Universidad Espíritu Santo, Samborondón 092301, Ecuador; 4 Instituto Nacional de Biodiversidad (INABIO), Quito 170505, Ecuador; 5 Institute of Integrative Biology, ETH, 8092 Zurich, Switzerland; 6 Facultad de Ciencias, Universidad Austral de Chile, Campus Isla Teja, Valdivia 5110566, Chile; 7 Departamento de Botánica, Facultad de Ciencias Naturales y Oceanográficas, Universidad de Concepción, Concepción 7800003, Chile; 8 Laboratorio de Invasiones Biológicas (LIB), Facultad de Ciencias Naturales y Oceanográficas, Universidad de Concepción, Concepción 7800003, Chile; 9 Laboratory for Research in Environmental Sciencies (LARES), Facultad de Ciencias Agronomicas, Universidad de Chile, Santiago 8820000, Chile; 10 Cape Horn Investigation Center (CHIC), Punta Arenas 62004100, Chile

**Keywords:** Andean mountains, biological invasions, climatic niche, elevation, elevational limit, roadsides

## Abstract

Alien plant species are colonizing high-elevation areas along roadsides. In this study, we evaluated whether the distributions of alien plants in the central Chilean mountains have reached climatic equilibrium (i.e., upper distribution limits consistent with their climatic requirements). First, we evaluated whether the upper elevational limits of alien plants changed between 2008 and 2018 based on the Mountain Invasion Research Network (MIREN) database. Second, we compared the observed upper elevational limits with the upper limits predicted by each species’ global climatic niche. On average across species, the upper elevation limit did not change between 2008 and 2018. However, most species maintained the same limit or shifted downward, while only 23% of the species shifted upwards. This lack of change does not mean that the species’ distributions are in equilibrium with the climate, because the observed upper limit was lower than the limit predicted by the global niche model for 87% of species. Our results suggest that alien species in this study region may not only be climate-limited, but could also be limited by other local-scale factors, such as seed dispersal, intermittent disturbance rates, soil type and biotic interactions.

## 1. Introduction

A well-documented pattern in invasion ecology is the significant decrease in alien plant diversity with elevation [1,2,3]. The overall temperature and the duration of the growing season, both decreasing with elevation, are regarded as the main explanatory factors for this pattern, suggesting that higher elevations act as environmental filters to limit the upward range expansion of alien plants [4]. Nonetheless, studies show that alien plants are actively expanding to higher elevations, thus opening questions about which those species can surmount this environmental filter [5,6,7,8]. Field studies conducted across different altitudinal gradients of the world (e.g., [1,9]), have shown that alien plant species that reach the highest elevations constitute a subset of the entire alien plant assemblage that occurs at lower elevations, acting as “climatic generalists” [1,10]. This pattern reinforces the idea that environmental conditions at high elevations are an important filter that only a subset of species can overcome.

Mountain ecosystems are very important because, despite representing only 27% of the Earth’s land surface, they harbor ca. 23% of the world’s forest and 25% of all terrestrial biodiversity, with high endemism rates [11,12,13]. The spread of alien species into mountain ecosystems can threaten the biodiversity of native species as well as affect the economy and human health [7,14,15]. Examining whether alien plants have attained climatic equilibrium (i.e., upper distribution limits consistent with their climatic requirements) might, therefore, reveal information relevant to managing such invasions and improving our capacity to predict future expansion in mountainous environments in the context of climatic change.

Niche theory establishes that species will occupy sites within environmental gradients with conditions suitable for their survival and reproduction (e.g., climatic requirements). The maximum elevation attained by one species may be explained mostly by its climatic niche [16]. If one alien plant has reached its climatic equilibrium, we expect that (i) its upper elevation limit will not change over time [17,18] and (ii) its upper elevation limit will match the that predicted from the climatic niche found in its global distribution [19].

Mountain roadsides can be used as natural experiments to test whether alien plant species attain their climatic equilibria; they constitute human-induced, disturbed habitats [20] that facilitate alien plant colonization and expansion [21,22,23,24]. Roads are regarded as corridors for the movement of species because (i) seed dispersal is facilitated by human and vehicular movements [20,25,26,27,28], and (ii) given that the diversity of native species tends to be low on the verges of roads [17,24], we expect competitive pressures to be reduced in these disturbed habitats. In summary, with seed dispersal facilitation and competitive pressures diminished, species along mountain roads could be more likely to attain the upper elevation expected from their climatic niches.

There are attributes other than climatic requirements that could modulate the expansion of alien plants to higher elevations. For example, species with low dispersal ability (e.g., gravity-dispersed seeds), can be far from equilibrium in relation to other species whose seeds are dispersed by animals or wind [16,29,30]. The inability to attain equilibrium can also just be a matter of time, since species with long residence times could attain climatic equilibrium sooner than those with shorter residence times [31,32]. Additionally, because annual species complete their life cycle yearly, they might be more likely to track their climatic niche and reach climatic equilibrium on mountains relative to perennial plant species, which delay the time to first reproduction.

The aim of this study was to evaluate whether the upper elevation limit (UEL) of alien plant species living in the mountains of central Chile (latitude 33° S) is consistent with the upper elevation limit predicted by their global climatic niches (i.e., climatic equilibrium). For this, we used two different approaches. First, we examined whether the observed UEL of alien plant species changed over a 10-year period from 2008 to 2018. If alien plant species were in climatic equilibria, and given the long residence time of many alien plants in Chile [33], we expected no significant UEL changes. Second, for a different set of alien plants existing along the altitudinal gradient obtained during 2018, we compared the observed UELs with those predicted from global climatic niches. If alien plant species are in climatic equilibrium, we expect a positive and significant relationship (with a slope not significantly different from 1) between the observed and predicted UEL. We also checked if differences in UEL between species are related to other plant attributes, such as dispersal ability, life span and residence time.

## 2. Results

### 2.1. Upper Elevational Limit (UEL) Changes over Time

**Figure 1:**
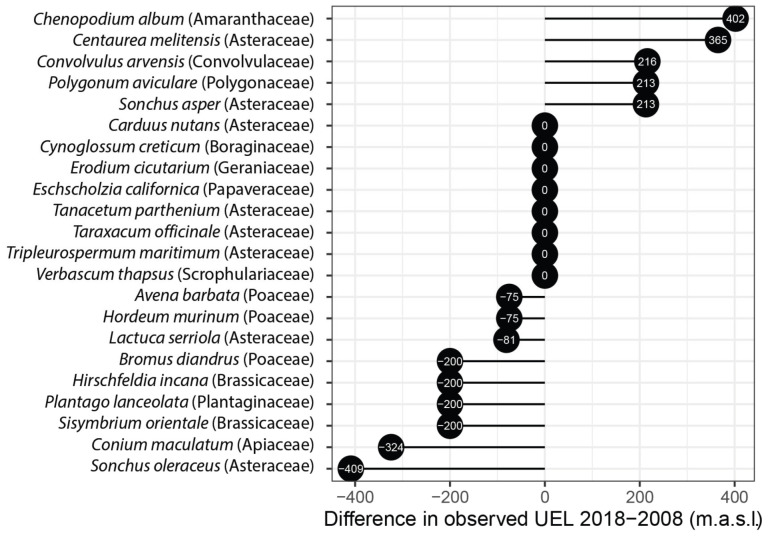
Temporal changes in the observed UEL (in meters) of 22 alien species recorded along an elevation gradient between 2008 and 2018 in the study area of central Chile (33°).

For this analysis, we used alien plant species that were observed along an elevation gradient (two mountain roads) in both 2008 and 2018 (*n* = 22). Globally, the UEL did not change between 2008 and 2018 (Wilcoxon test, W = 219.50, *p* = 0.60). At the species level, we observed that five species (23%) increased their UEL by more than 200 m, nine species (41%) shifted downwards in a range from 75 to 409 m and only eight species (36%) did not change their UEL over the period of 10 years (Figure 1).

### 2.2. Assessing If Species’ UELs Are in Equilibrium with Climate

**Figure 2:**
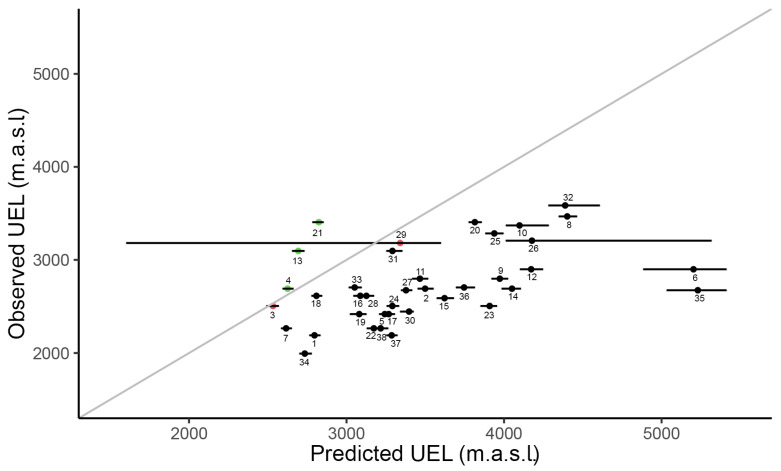
Comparison between observed upper elevation limits (UELs) along two mountain roads in Central Chile (33° S) and UELs predicted from the species’ global climatic niches. The grey line represents the x = y line (slope = 1). Points represent species (with ID) and horizontal black lines represent the 95% confidence intervals of the predicted UELs. Red points represent species in climatic equilibrium. Black points represent species that occur at a lower elevation than the predicted UEL. Green points represent species that occur at a higher elevation than the predicted UEL. 1: *Anagallis arvensis*, 2: *Anthemis cotula*, 3: *Avena barbata*, 4: *Bromus diandrus*, 5: *Bromus scoparius*, 6: *Carduus nutans*, 7: *Centaurea melitensis*, 8: *Cerastium arvense*, 9: *Chamomilla suaveolens*, 10: *Chenopodium album*, 11: *Conium maculatum*, 12: *Convolvulus arvensis*, 13: *Cynoglossum creticum*, 14: *Echium vulgare*, 15: *Erodium cicutarium*, 16: *Eschscholzia californica*, 17: *Galium aparine*, 18: *Hirschfeldia incana*, 19: *Hordeum murinum,* 20: *Lactuca serriola*, 21: *Lactuca virosa*, 22: *Marrubium vulgare*, 23: *Onopordum acanthium*, 24: *Plantago lanceolata*, 25: *Polygonum aviculare*, 26: *Rumex acetosella*, 27: *Rumex crispus*, 28: *Sisymbrium orientale*, 29: *Sonchus asper*, 30: *Sonchus oleraceus*, 31: *Tanacetum parthenium*, 32: *Taraxacum officinale*, 33: *Tragopogon porrifolius*, 34: *Trifolium glomeratum*, 35: *Tripleurospermum maritimum*, 36: *Verbascum thapsus*, 37: *Veronica persica* and 38: *Vulpia myuros*.

We obtained a positive and significant relationship between the observed UELs in the study region and the UELs that were predicted from species’ global niches across species (r^2^ = 0.44, t =3.02, d.f. = 36, *p*-value = 0.0045, Figure 2); however, the slope of this relationship was significantly lower than 1 (b = 0.27, t = 7.96, *p*-value < 0.0001), suggesting that, on average, alien species have not attained climatic equilibrium and the upper distribution limits did not match their climatic tolerances. We found that, in the great majority of species (87%), the observed UEL was lower than the predicted UEL (Figure 2). In contrast, in three species (8%), the observed UEL was higher than the predicted UEL, and in two species (5%), the observed UEL was within the 95% confidence interval (Figure 2). The climatic niches on gridded environmental spaces for the studied species are shown in Figures S1–S5. The observed and predicted UEL values with confidence intervals are presented in Table S1.

### 2.3. UEL and Species Attributes

We did not find a significant relationship between the residence time, life span or dispersal ability and elevational changes between 2008 and 2018 (Table 1 (a)). Similarly, the standardized difference between the observed and predicted UEL among species had no relationship with the residence time, life span or dispersal ability (Table 1 (b)).

## 3. Discussion

We tested whether alien plant species in our study region in the Andes of central Chile have attained their climatic equilibrium by (i) comparing the change in the upper elevational limit (UEL) of their distribution between 2008 and 2018 and by (ii) comparing the observed UEL with the predicted UEL from the climatic niche of each species at a global scale. Our results demonstrated that over this 10-year period, relatively few species (36%) showed stable elevational limits. The rest of the species changed elevation either upward or downward in the same period. Additionally, despite the residence time of the analyzed alien species being 80–100 years on average [33], comparisons using climatic niche models revealed that 87% of species had not attained their climatic equilibrium, suggesting room for further upward expansion.

### 3.1. Temporal Changes

Although we did not detect significant altitudinal changes over a period of 10 years on average across species (2008–2018), at the species level, we determined that eight species conserved their upper elevation limits, five species expanded their upper elevation limits and nine species reduced their upper elevation limits. We observed that all three Poaceae species moved downward by approx. 70–400 m in this 10-year period (Figure 1). This downward shift in a set of grasses that are regarded as very invasive across diverse ecosystems [34] suggests that they are intolerant to the increased drought that had affected the study area during this period. We hypothesize that the former limit was maintained by propagules produced at lower elevations and that now cannot be so readily produced due to the drought. Our study area in central Chile has experienced an uninterrupted sequence of dry years since 2010, with mean rainfall deficits of 20–40% [35].

In the period of ten years, only 23% of the species expanded their distribution to a higher elevation. One explanation is that, in our study area, above 3000 m a.s.l, the mountain roads became precarious and the transit of vehicles is reduced significantly [36,37] (Goncalves pers. obs.). Even having climatic conditions that allow some alien species to live there, the paucity of propagules would hinder their arrival and establishment at these elevations. Additionally, plant establishment should decrease because anthropogenic disturbance from road maintenance and vegetation mowing is reduced along such poorly used roads [37,38,39]. Recent studies in the Andes of southern Chile demonstrated that anthropogenic disturbances are the most important driver of the richness and abundance of alien species [38].

### 3.2. Mismatch between Observed and Predicted UEL

Our results indicated that, in general, there is a mismatch between the observed UELs in our study region and the UELs predicted from species’ global climatic niches. First, we detected that the slope of the relationship between these two variables was significantly less than 1. Second, in most species (87%), the predicted UEL was significantly higher than the observed UEL. This pattern was maintained in those species that moved downward from 2008–2018. That is, in a period of ten years, their altitude was always below the predicted upper elevation limit. Thus, the absence of a climatic equilibrium seems to be the norm for this set of alien species. If other attributes, such as residence time and functional traits (dispersal mode and life span), did not explain the expansion to higher elevations, what are the limiting factors that prevent the colonization of suitable habitats at higher elevation? We believe that it is important and necessary to examine fine-scale limiting factors (not evaluated in this study), such as soil type variation, intermittent roadside disturbance, or negative biotic interactions [36,37,38,40]. Manipulative experiments could be designed to examine these potential limiting factors.

Genetic bottlenecks could also explain the fact that most alien species did not reach climatic equilibrium. Typically, introduced populations are expected to pass through genetic bottlenecks, with allelic diversity being lost through both founder effects and drift [41]; it is possible that the populations introduced to our study area do not represent all of the genetic diversity included in the global niche models, reflecting a shorter elevational range in the gradient. Additionally, alien species probably had to become adapted to lowland Chilean conditions before expanding into the mountains. The severity of genetic loss through bottlenecks depends on several factors, including the introduction history (i.e., the quantity and sources of introduced propagules) [42]. Unfortunately, we do not have information about the introduction history or evidence of multiple introductions of each alien species that could temper bottleneck effects. Therefore, we cannot exclude the bottleneck hypothesis as a limiting factor for the species range limit.

The cases of *Bromus diandrus*, *Cynoglossum creticum* and *Lactuca virosa* are particularly interesting, because these three species were the only ones for which the observed UEL was higher than the predicted UEL. One possible explanation may be plant–plant facilitation, a well-studied phenomenon in our study area that is mediated by native cushion plants [43]. However, we observed no cushion plants on the roadsides. Alternatively, individuals present on roadsides may constitute sink populations [44]. The population density of these three species was extremely low (<2 ind/100 m^2^) and reproduction was not observed (E. Golcalves, unpublished data) at their observed upper elevation limit. Thus, it seems likely that individuals observed at high elevations could be migrants from lower source populations; demographically, they can germinate, survive and grow, but they are unable to reproduce and maintain viable local populations [18,45,46]. Population studies at the species’ distribution limits may shed light on such source–sink population dynamics.

For the two species for which were in climatic equilibrium (*Avena barbata*, *Sonchus asper*), the implications may be important. If their niches are conserved, then we do not expect expansion to higher elevations, although with climate change, these species probably could expand to higher elevations by tracking their niche requirements. Assuming that maximal elevation is governed mainly by climate, these two species could serve as sentinels to monitor the further expansion of alien species in response to climatic change. Experiments and monitoring would be needed to test whether these species shift their niches, moving higher over time.

To our knowledge, the approach of estimating the UELs of alien species in mountains from their global climatic niches has not been used previously. Given that we focused on global climatic niches, this method was conducted at a large spatial scale, and was thus insensitive to other finer-scale factors that also contribute to shaping elevation patterns (e.g., soil type, biotic interactions and microclimate generated by disturbance changes, topography, or solar exposure). Our methodology could be useful as a first approximation to study plant invasions on mountains, providing a simple statistical method based on resampling to test for concordance between observations and predictions.

We have shown that most alien species in central Chile have not reached climatic equilibrium. It is crucial to carry out long-term monitoring of alien species in mountain ecosystems to estimate the dispersal rates beyond the current limits over time and to examine the extent to which climatic change will modify the expansion of alien plants into mountain ecosystems.

## 4. Materials and Methods

### 4.1. Study Area

The study area is located in the Andes of central Chile (33° S; 70° W). The climate is Mediterranean with wet, cold winters and dry, warm summers [47,48]. During winter, precipitation occurs mostly as snow, although hail and snow can also occur occasionally during the summer at higher elevations [49]. Snow cover remains present from 3 to 5 months depending on elevation and solar exposure [50]. At 2000 m elevation, the average annual temperature is ca. 6.5 °C, and at 3150 m, it decreases to 3 °C [47]). At 3200 m elevation, the annual precipitation is ca. 943 mm, mostly as snow from May to October [51]. The average adiabatic lapse rate is approx. 6.1 °C/km, with a seasonal variation of 4 °C/km in summer and 7 °C/km in winter [47].

### 4.2. Assessing Species Upper Elevational Limit (UEL) Changes over Time

To assess UEL changes between 2008 and 2018, we used a dataset collected following the Mountain Invasion Research Network (MIREN; https://www.mountaininvasions.org/, accessed on 3 August 2021) sampling protocol [52]. Briefly, during November 2007–March 2008 and November 2017–March 2018, the presence of all alien plant species along two roads in the study area, one road from Farellones to La Parva (1900 m to 3600 m.a.s.l.) and the other from Farellones to Valle Nevado (2400 to 3500 m.a.s.l.), was determined. With every 100 m gain in elevation, a transect was laid, consisting of one 2 × 50 m plot parallel to the road and another two 2 × 50 m plots perpendicular to the road, arranged as a “T” [52]. For the purposes of this study, we used only the plots parallel to the roads, from which we obtained the UEL for each species in 2008 and 2018. Then, we compared the observed UELs across alien plants between years by using a non-parametric Wilcoxon test in R version 4.1.0. Finally, we used a linear model to assess whether the change in elevation between 2008 and 2018 was related to residence time, life span (perennial, biennial or annual) and dispersal mode (wind, animal or non-assisted), as determined in [33].

### 4.3. Assessing if Species’ UELs Are in Climatic Equilibrium

**Figure 3:**
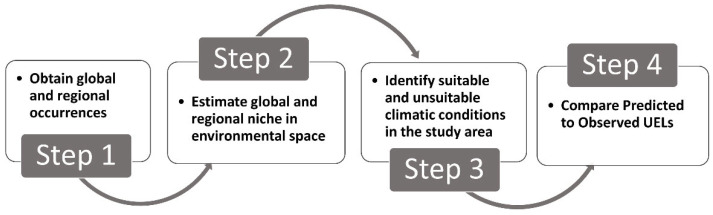
Workflow diagram used to estimate the predicted upper elevational limit (UEL) for a set of alien species in mountain ecosystems of central Chile.

To evaluate whether the observed UELs of species are in equilibrium with the climate, we compared the observed UEL with a predicted UEL estimated from the global climatic niche. To generate the predicted UELs, we proceeded in four steps, as follows (see Figure 3).

Step 1. Obtain global and regional occurrences

Global occurrences for the alien plants recorded along elevation gradients were obtained from the Global Biodiversity Information Facility database (GBIF.org– https://doi.org/10.15468/dl.tttyn6, accessed on 3 August 2021). We selected only occurrences in which the coordinate error was less than 1 km. Regional occurrences were obtained from the MIREN 2018 database, selecting only roadside plots, with 12 plots from the first road (Farellones to La Parva: 1900 m to 3600 m.a.s.l.) and 9 plots from the second road (Farellones to Valle Nevado: 2400 to 3500 m.a.s.l.), a total of 21 plots.

**Figure 4:**
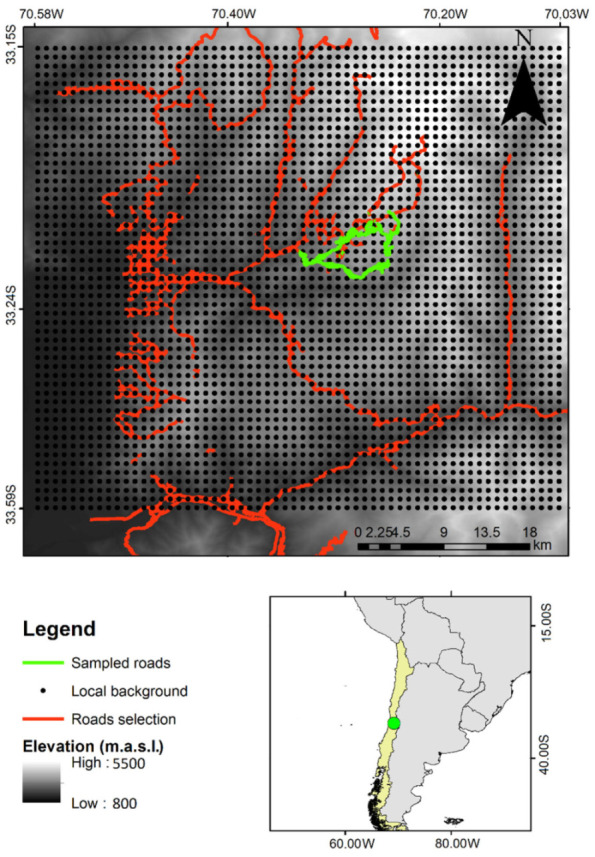
Study area depicting regularly sampled points to depict the regional climatic space. Local background is represented by 3180 geo-referenced points at a resolution f 30 arc sec (~1 km^2^). The road network is shown in red and the mountain roads selected for this study are shown in green. The grayscale gradient depicts elevation (m a.s.l.).

Additionally, to obtain a more detailed picture of the regional elevation distribution of these species, we conducted a field survey, sampling the same two mountain roads from the MIREN database (Figure 4). Vegetation sampling was conducted between November 2017 and March 2018 using 100 m^2^ plots parallel to mountain roads, laid out every 50 m in elevation. On the first road (from Farellones to La Parva: 2000 to 3500 m.a.s.l.), we sampled 30 plots, and on the second road (from Farellones to Valle Nevado: 2400 to 3500 m.a.s.l.), we sampled 24 plots, thus giving a total of 54 plots. We registered the presence of species in every plot. The numbers of global and regional occurrences used for each species are presented in Table S2 of the Supporting Information. the elevation and coordinates from the field survey plots and MIREN database plots are presented in Table S3 of the Supporting Information.

Step 2. Estimate global and regional niche in environmental space

For the construction of the niche models, we used the framework proposed by [53]. In brief, this method describes and compares niches in a gridded environmental space, where each cell corresponds to a set of environmental conditions. For each species, four regions are depicted in the grid: (a) the global environmental space, e.g., the climatic conditions existing worldwide; (b) the species’ global niche, constructed with global occurrences; (c) regional environmental space, e.g., the climatic conditions existing in the study region; and (d) regional niche constructed with local occurrences. For the purposes of this study, we focused only on the global niche and the regional environmental space (explained below).

To represent the global environmental space, we generated 10,000 geo-referenced random points worldwide (except for Antarctica) and extracted the values of 19 climatic variables obtained from Worldclim 2.1 at a topoclimate scale: 30 arc sec ~1 km^2^ resolution [54]. For the representation of the regional climatic environmental space (see Figure 4), we delimited the study area as a rectangle including the two sampled roadsides located approximately in its center, encompassing a total area of 2862 km^2^ (rectangle vertices coordinates: 70.58 W, 33.59 S, 70.03 W and 33.15 S). Then, we divided this study area into 0.9 km × 0.9 km pixels following the raster resolution in Worldclim 30 arc sec (approx. 1 km^2^) and generated one geo-referenced point within each of the pixels, obtaining 3180 total points. This is a systematic sampling design to encompass the totality of the climatic conditions of the study area. For each of the 3180 geo-referenced points, we extracted the values of 19 climatic variables obtained from Worldclim and depicted them on the environmental grid [53]. For PCA construction, we used the dudi.pca function from the “ade4” package [55], and for the niche visualization, we used the “ecospat” package [56] in R version 4.1.0.

Step 3. Identify suitable and unsuitable climatic conditions in the study area

**Figure 5:**
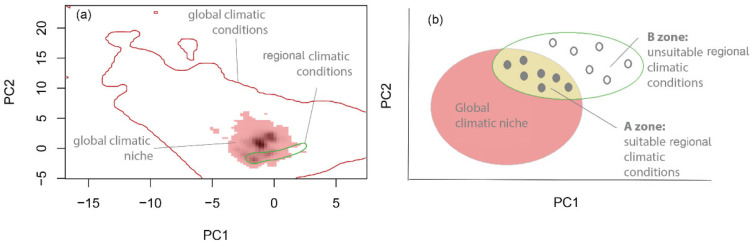
Diagrammatic representation of the environmental grid [53] to identify suitable and unsuitable habitats. (**a**) PCA with global climatic niches, intersected with the regional scale (study area in central Chile) climate niche for *Tragopogon porrifolius* as an example. (**b**) Identification of suitable (black points) and unsuitable conditions (white points) for the species in the study area in Chile.

To identify suitable and unsuitable conditions, we intersected the species’ global climatic niche with the climatic regional environmental space. First, we delimited the global climatic niche, depicted within axes 1 and 2 of the PCA (Figure 5), selecting 99% of the surface and leaving out the most extreme 1% of the niche surface. Within the same PCA space, we represented the climatic regional environmental space, extracting the climatic variables for each of the 3180 points selected within the study area (Figure 4). The intersection between the global climatic niche and the regional climatic environmental space was used to define suitable and unsuitable climatic conditions. The climatic regional points within a species’ global climatic niche were assumed to be climatically suitable (zone A in Figure 5), while those outside the global climatic niche, but within the regional environmental climatic space, were assumed unsuitable (zone B in Figure 5). For each of the 3180 points in the A and B zones (Figure 5), we extracted the elevation (m a.s.l.) with the digital elevation model at 30 m resolution and global SRTM [57].

Step 4. Compare predicted to observed UELs

To evaluate whether the species’ observed UELs were in climatic equilibrium, we compared the observed values with the predicted UELs obtained from global climatic niches. To estimate the predicted UEL, we used Hiussman–Olff–Fresco models (HOF) [58]. HOF curves are logistic regression statistical models that relate presence/absence data to gradient data; in essence, these models represent species’ responses along environmental gradients [59]. In our study, the environmental gradient was elevation; presences (1) were obtained within the A zone with suitable climatic conditions, while absences (0) were obtained within the B zone with unsuitable climatic conditions (See Figure 5). The HOF curves presented seven alternative statistical models for each species; the best was selected using the Akaike information criterion (AIC).

One property of HOF models is that they provide a series of parameters for the species distribution curve along environmental gradients (in our case, elevation). We selected the outer border (OB) as the predicted UEL, defined as the elevation at which the occurrence probabilities reached exp(−2) relative to the highest estimated response value [60]. To estimate the variability of the predicted UEL, we constructed 100 HOF models for each species. Specifically, for each HOF model, we randomly extracted 25% of 1’s in the suitable zone and 25% of 0’s in unsuitable zones (Figure 5b). We took this approach, instead to use a subsample 25% from the total data (0,1 together) because in some cases the number of 1’s was very low and in such cases HOF curves lose statistical confidence [59].

For every HOF model, we extracted the outer border Consequently, we obtained a sample of 100 outer border values, which allowed the construction of its sampling distribution; from these data, we estimated a mean outer border and its 95% confidence interval. If the observed UEL was within the 95% confidence interval, then we accepted the hypothesis that the species had reached climatic equilibrium; otherwise, we rejected the hypothesis of climatic equilibrium.

### 4.4. UEL and Species Attributes

We related species ecological traits (e.g., life span or dispersal mode) and residence time with the standardized difference between the observed UEL (UELo) and predicted UEL (UELp) (∆UEL = $\frac{\left|{\mathrm{UEL}}_{O}-{\mathrm{UEL}}_{p}\right|}{\mathrm{UELp}}$) using a linear model after checking for data normality and heteroscedasticity assumptions. These analyses were conducted in R version 4.1.0. The ecological traits for the studied species were obtained from [33] and are presented in Table S4.

## Figures and Tables

**Table 1:** ANOVA test results for the relations between residence time, dispersal mode (animal, wind or unassisted) and life span (annual, biennial or perennial) with two response variables: (a) observed UEL difference from 2018–2008 and (b) standardized difference between the observed and predicted UEL.

Response Variable	Variable	Df	Sum Sq	Mean Sq	F Value	*p*-Value
(a) Observed UEL difference between years (2018–2008)	Residence time	1	13	13	0.0002	0.9878
	Dispersal mode	2	87,625	43,812	0.8483	0.4505
	Life span	2	77,297	38,648	0.7483	0.4925
	Residuals	13	671,395	51,646		
(b) Standardized difference between observed and predicted UEL	Residence time	1	0.00522	0.0052192	0.2572	0.6162
	Dispersal mode	2	0.01949	0.0097462	0.4802	0.6238
	Life span	2	0.00644	0.0032198	0.1586	0.8541
	Residuals	27	0.54799	0.0202959		

## Data Availability

Regional occurrence data and the scripts used in analysis will be uploaded in a Dryad public repository. The global occurrence data are already available at https://doi.org/10.15468/dl.tttyn6 (accessed on 3 August 2021).

## Supplementary Materials

The following supporting information can be downloaded at: https://www.mdpi.com/article/10.3390/plants11182345/s1, Figures S1–S5: PCA-env for the studied species. Red line represents global climatic background conditions and green line represents climatic background conditions in the study area. Red pixels represents global climatic niche. Gray–black gradient represents the most occupied global climatic conditions by species. Table S1: Comparison between observed and expected UEL for a set of species observed during 2018. Expected UEL was estimated by the average outer border (OB) obtained from 100 HOF curves. Table S2. Number of global and regional occurrences used for each species for niche estimation in environmental space. Table S3: Elevation and coordinates from field survey plots and MIREN database plots (LP: road from Farellones to La Parva, VN: road from Farellones to Valle Nevado). Table S4: Ecological traits and factors of 38 exotic species in the study region
