# Supplementary material for: The Upper Range Limit of Alien Plants Is Not in Equilibrium with Climate in the Andes of Central Chile
Source: Plants (Basel). 2022 Sep 8;11(18):2345. doi: 10.3390/plants11182345 (PMC9501811; doi:10.3390/plants11182345)
Supplement: Supplementary file 1 [file plants-11-02345-s001.zip › plants-1866572-supplementary.pdf]

# Supplementary Material

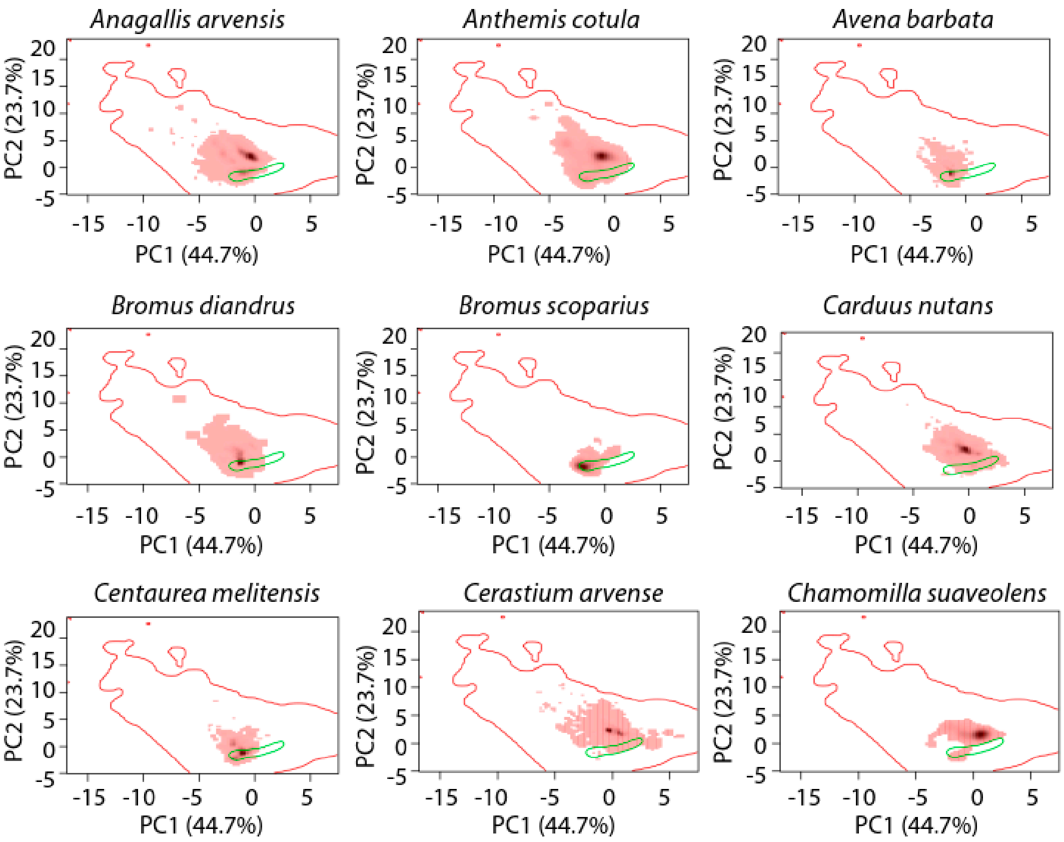


Figure S1: PCA-env for study species. Red line represents global climatic background conditions and green line represents climatic background conditions in study area  Red pixels represents global climatic niche. The grey-black gradient represents the most occupied global climatic conditions by species.

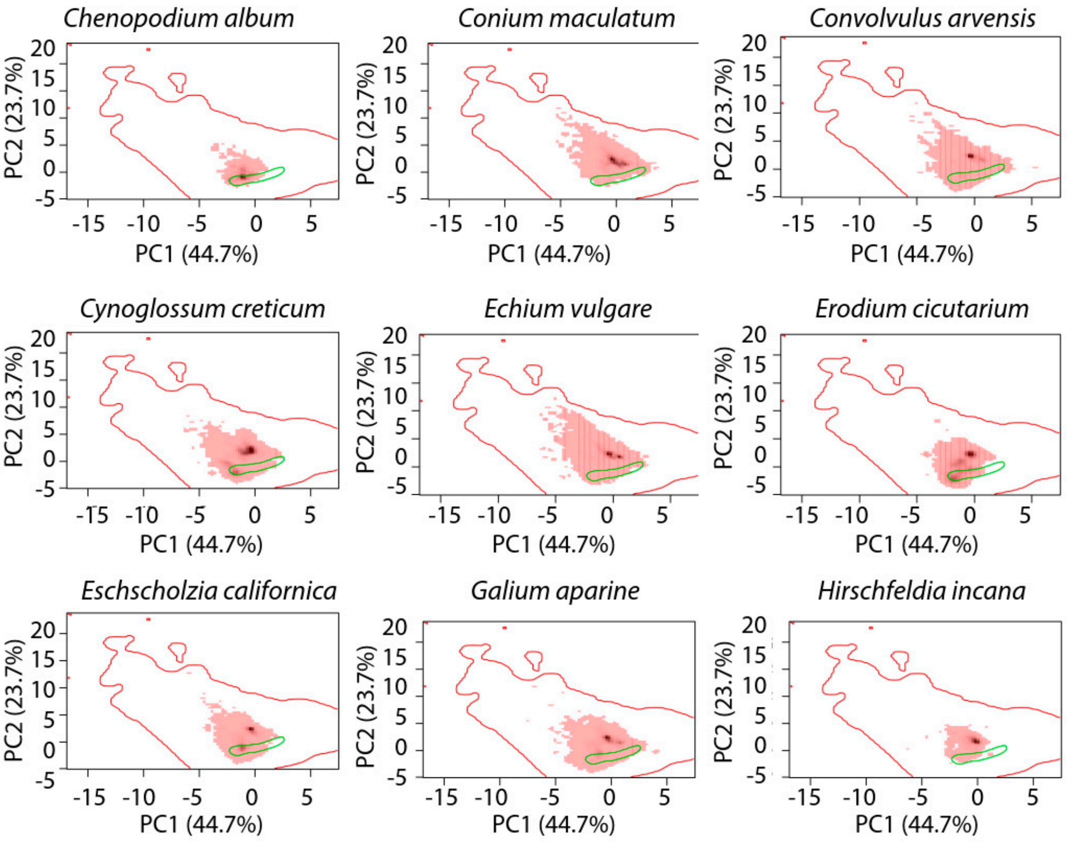


Figure S2: PCA-env for study species. Red line represents global climatic background conditions and green line represents climatic background conditions in study area  Red pixels represents global climatic niche. The grey-black gradient represents the most occupied global climatic conditions by species.

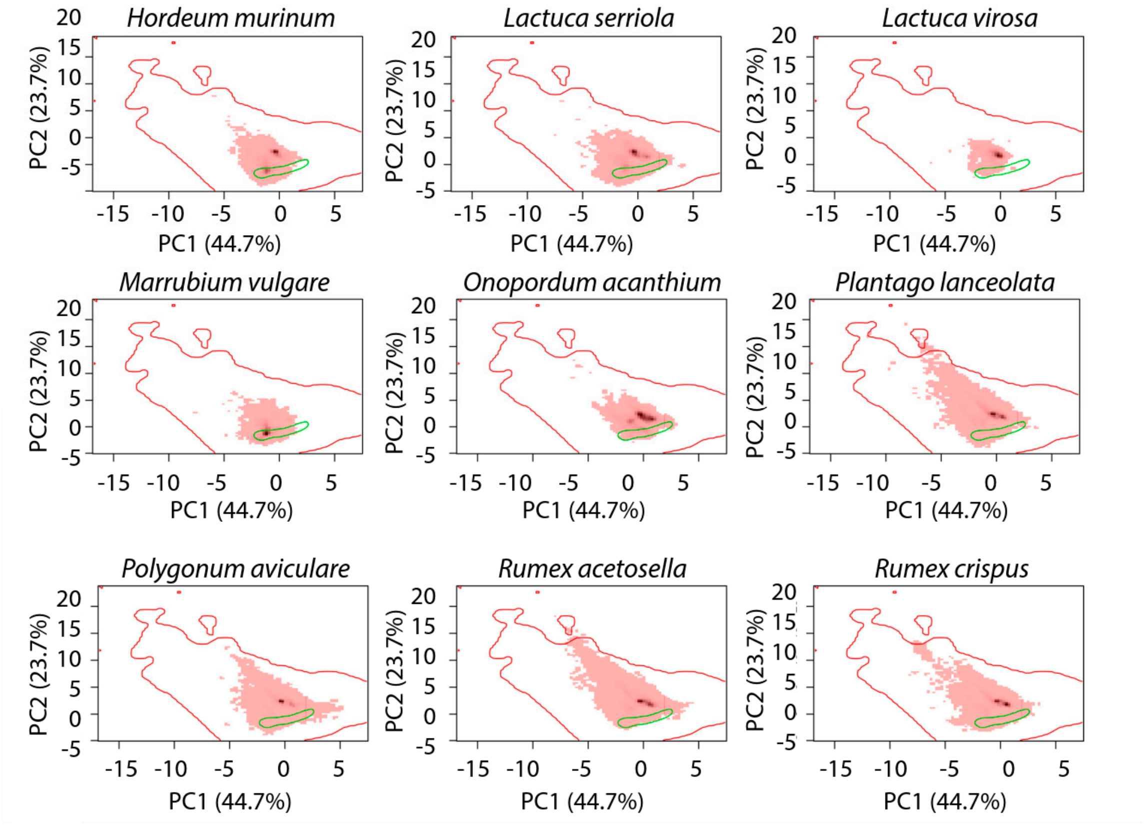


Figure S3: PCA-env for study species. Red line represents global climatic background conditions and green line represents climatic background conditions in study area  Red pixels represents global climatic niche. The grey-black gradient represents the most occupied global climatic conditions by species.

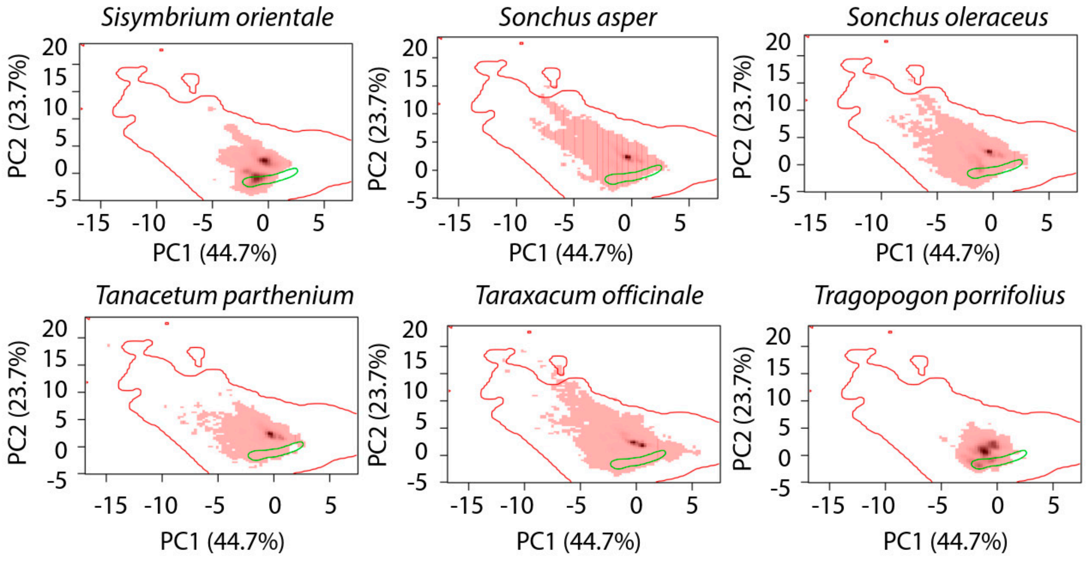


Figure S4: PCA-env for study species. Red line represents global climatic background conditions and green line represents climatic background conditions in study area Red pixels represents global climatic niche. The grey-black gradient represents the most occupied global climatic conditions by species.

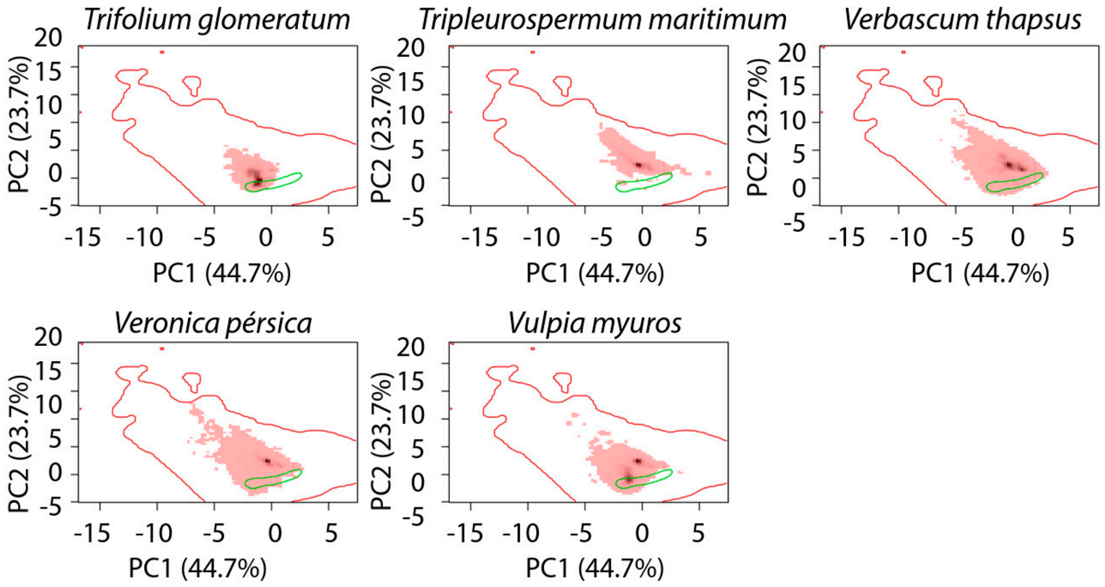


Figure S5: PCA-env for study species. Red line represents global climatic background conditions and green line represents climatic background conditions in study area Red pixels represents global climatic niche. The grey-black gradient represents the most occupied global climatic conditions by species.

Table S1: Comparison between observed and expected UEL for a set of species observed during 2018. Expected UEL was estimated by the average Outer Border (OB) obtained from 100 HOF curves. The quantile 0.025 and 0.975 is presented in the Table (95% CI), and were obtained from the sampling distribution of the 100 values of OB. Delta values represent the difference in meters between the Observed and mean predicted UEL. In (*) the species which are in climatic equilibrium; (**) species which observed UEL is greater than expected by climatic niche.

| **ID** | **Species** | **Family** | **Observed UEL** | **Mean Predicted UEL** | **Quantile 0.025** | **Quantile 0.975** | **Delta** |
|---|---|---|---|---|---|---|---|
| 1 | *Anagallis arvensis* | Primulaceae | 2190 | 2796.58 | 2763.30 | 2834.94 | -606.58 |
| 2 | *Anthemis cotula* | Asteraceae | 2691 | 3498.94 | 3450.75 | 3553.30 | -807.94 |
| 3 | *Avena barbata** | Poaceae | *2505* | *2533.19* | *2490.56* | *2570.91* | *-28.19* |
| 4 | *Bromus diandrus*** | Poaceae | 2691 | 2625.95 | 2593.41 | 2663.78 | 65.05 |
| 5 | *Bromus scoparius* | Poaceae | 2418 | 3242.69 | 3206.48 | 3292.67 | -824.69 |
| 6 | *Carduus nutans* | Asteraceae | 2899 | 5202.89 | 4883.28 | 5413.00 | -2303.89 |
| 7 | *Centaurea melitensis* | Asteraceae | 2265 | 2616.32 | 2584.08 | 2653.12 | -351.32 |
| 8 | *Cerastium arvense* | Caryophyllaceae | 3467 | 4401.18 | 4346.47 | 4464.10 | -934.18 |
| 9 | *Chamomilla suaveolens* | Asteraceae | 2798 | 3973.00 | 3919.76 | 4026.28 | -1175.00 |
| 10 | *Chenopodium álbum* | Amaranthaceae | 3370 | 4098.15 | 4010.97 | 4284.31 | -728.15 |
| 11 | *Conium maculatum* | Apiaceae | 2798 | 3465.75 | 3416.21 | 3518.94 | -667.75 |
| 12 | *Convolvulus arvensis* | Convolvulaceae | 2899 | 4172.05 | 4100.76 | 4248.07 | -1273.05 |
| 13 | *Cynoglossum creticum*** | Boraginaceae | 3096 | 2693.30 | 2653.97 | 2733.52 | 402.70 |
| 14 | *Echium vulgare* | Boraginaceae | 2691 | 4050.44 | 3984.22 | 4107.37 | -1359.44 |
| 15 | *Erodium cicutarium* | Geraniaceae | 2589 | 3622.10 | 3572.01 | 3682.20 | -1033.10 |
| 16 | *Eschscholzia califórnica* | Papaveraceae | 2614 | 3087.32 | 3042.99 | 3127.17 | -473.32 |
| 17 | *Galium aparine* | Rubiaceae | 2418 | 3267.92 | 3224.59 | 3307.81 | -849.92 |
| 18 | *Hirschfeldia incana* | Brassicaceae | 2614 | 2808.73 | 2776.77 | 2846.00 | -194.73 |
| 19 | *Hordeum murinum* | Poaceae | 2418 | 3080.63 | 3020.91 | 3127.04 | -662.63 |
| 20 | *Lactuca serriola* | Asteraceae | 3405 | 3814.59 | 3774.67 | 3858.81 | -409.59 |
| 21 | *Lactuca virosa*** | Asteraceae | 3405 | 2823.96 | 2782.64 | 2855.10 | 581.04 |

| | | | | | | | |
|---|---|---|---|---|---|---|---|
| 22 | *Marrubium vulgare* | Lamiaceae | 2265 | 3173.28 | 3127.89 | 3223.03 | -908.28 |
| 23 | *Onopordum acanthium* | Asteraceae | 2505 | 3908.37 | 3850.14 | 3956.14 | -1403.37 |
| 24 | *Plantago lanceolata* | Plantaginaceae | 2505 | 3290.87 | 3254.16 | 3334.44 | -785.87 |
| 25 | *Polygonum aviculare* | Polygonaceae | 3284 | 3938.04 | 3880.76 | 3996.94 | -654.04 |
| 26 | *Rumex acetosella* | Polygonaceae | 3206 | 4176.92 | 4010.41 | 5318.00 | -970.92 |
| 27 | *Rumex crispus* | Polygonaceae | 2674 | 3377.56 | 3345.28 | 3417.54 | -703.56 |
| 28 | *Sisymbrium orientale* | Brassicaceae | 2614 | 3125.98 | 3085.29 | 3175.62 | -511.98 |
| 29 | *Sonchus asper** | Asteraceae | *3181* | *3340.24* | *1601.28* | *3600.25* | *-159.24* |
| 30 | *Sonchus oleraceus* | Asteraceae | 2445 | 3396.89 | 3339.55 | 3426.50 | -951.89 |
| 31 | *Tanacetum parthenium* | Asteraceae | 3096 | 3291.47 | 3252.35 | 3355.19 | -195.47 |
| 32 | *Taraxacum officinale* | Asteraceae | 3585 | 4388.00 | 4281.17 | 4608.61 | -803.00 |
| 33 | *Tragopogon porrifolius* | Asteraceae | 2704 | 3051.58 | 3013.62 | 3096.67 | -347.58 |
| 34 | *Trifolium glomeratum* | Fabaceae | 1994 | 2734.86 | 2700.15 | 2779.95 | -740.86 |
| 35 | *Tripleurospermum maritimum* | Asteraceae | 2674 | 5230.21 | 5032.00 | 5413.00 | -2556.21 |
| 36 | *Verbascum thapsus* | Scrophulariaceae | 2704 | 3746.14 | 3694.05 | 3815.60 | -1042.14 |
| 37 | *Veronica persica* | Scrophulariaceae | 2190 | 3286.52 | 3252.43 | 3324.02 | -1096.52 |
| 38 | *Vulpia myuros* | Poaceae | 2265 | 3216.55 | 3174.72 | 3265.55 | -951.55 |

Table S2: Number of Global and Regional occurrences used for each species for niche estimation in environmental space. For Regional occurrences we used data from MIREN database and field work data collected by authors during the study.

| Specie | Global data | Regional data (MIREN) | Regional data (field work) | Regional data total |
|---|---|---|---|---|
| *Anagallis arvensis* | 19857 | 1 | 0 | 1 |
| *Anthemis cotula* | 3485 | 0 | 4 | 4 |
| *Avena barbata* | 51497 | 14 | 11 | 25 |
| *Bromus diandrus* | 26550 | 4 | 13 | 17 |
| *Bromus scoparius* | 285 | 0 | 3 | 3 |
| *Carduus nutans* | 13507 | 6 | 5 | 11 |
| *Centaurea melitensis* | 9342 | 4 | 0 | 4 |
| *Cerastium arvense* | 28921 | 0 | 25 | 4 |
| *Chamomilla suaveolens* | 167 | 0 | 2 | 2 |
| *Chenopodium album* | 111833 | 1 | 34 | 35 |

| | | | | |
|---|---|---|---|---|
| *Conium maculatum* | 24311 | 2 | 13 | 15 |
| *Convolvulus arvensis* | 89919 | 10 | 26 | 36 |
| *Cynoglossum creticum* | 5809 | 10 | 24 | 34 |
| *Echium vulgare* | 82033 | 1 | 7 | 8 |
| *Erodium cicutarium* | 70388 | 21 | 12 | 33 |
| *Eschscholzia californica* | 39549 | 14 | 18 | 32 |
| *Galium aparine* | 220289 | 0 | 3 | 3 |
| *Hirschfeldia incana* | 8797 | 20 | 20 | 40 |
| *Hordeum murinum* | 56939 | 14 | 13 | 27 |
| *Lactuca serriola* | 60626 | 20 | 25 | 45 |
| *Lactuca virosa* | 2303 | 0 | 47 | 47 |
| *Marrubium vulgare* | 29708 | 6 | 0 | 6 |
| *Onopordum acanthium* | 12869 | 0 | 8 | 8 |
| *Plantago lanceolata* | 357358 | 1 | 11 | 12 |
| *Polygonum aviculare* | 112874 | 32 | 45 | 77 |
| *Rumex acetosella* | 159211 | 1 | 4 | 5 |
| *Rumex crispus* | 157020 | 4 | 9 | 13 |
| *Sisymbrium orientale* | 4626 | 16 | 24 | 40 |
| *Sonchus asper* | 96031 | 12 | 5 | 17 |
| *Sonchus oleraceus* | 115114 | 3 | 4 | 7 |
| *Tanacetum parthenium* | 29745 | 24 | 27 | 51 |
| *Taraxacum officinale* | 151247 | 42 | 86 | 126 |
| *Tragopogon porrifolius* | 7291 | 0 | 8 | 8 |
| *Trifolium glomeratum* | 9807 | 0 | 1 | 1 |
| *Tripleurospermum maritimum* | 14036 | 11 | 19 | 30 |
| *Verbascum thapsus* | 65923 | 4 | 13 | 17 |
| *Veronica persica* | 79079 | 1 | 0 | 1 |
| *Vulpia myuros* | 18985 | 2 | 0 | 2 |

Table S3: Elevation and coordinates from field survey plots and MIREN database plots (LP: road from Farellones to La Parva, VN: road from Farellones to Valle Nevado).

| Longitude | Latitude | Elevation (m.a.s.l.) | Road | Database |
|---|---|---|---|---|
| -33.3534 | -70.3289 | 1994 | LP | field survey |
| -33.3559 | -70.3276 | 2091 | LP | field survey |
| -33.3531 | -70.3270 | 2050 | LP | field survey |
| -33.3567 | -70.3262 | 2147 | LP | field survey |
| -33.3573 | -70.3250 | 2206 | LP | field survey |
| -33.3553 | -70.3201 | 2260 | LP | field survey |
| -33.3554 | -70.3185 | 2320 | LP | field survey |
| -33.3554 | -70.3180 | 2353 | LP | field survey |
| -33.3554 | -70.3185 | 2418 | LP | field survey |
| -33.3489 | -70.3079 | 2505 | LP | field survey |
| -33.3448 | -70.3019 | 2557 | LP | field survey |
| -33.3413 | -70.2944 | 2614 | LP | field survey |
| -33.3422 | -70.2946 | 2648 | LP | field survey |

| | | | | |
|---|---|---|---|---|
| -33.3389 | -70.2903 | 2691 | LP | field survey |
| -33.3363 | -70.2882 | 2754 | LP | field survey |
| -33.3351 | -70.2872 | 2798 | LP | field survey |
| -33.3324 | -70.2850 | 2858 | LP | field survey |
| -33.3292 | -70.2869 | 2899 | LP | field survey |
| -33.3301 | -70.2830 | 2951 | LP | field survey |
| -33.3286 | -70.2827 | 2999 | LP | field survey |
| -33.3268 | -70.2797 | 3059 | LP | field survey |
| -33.3280 | -70.2790 | 3096 | LP | field survey |
| -33.3311 | -70.2765 | 3139 | LP | field survey |
| -33.3298 | -70.2730 | 3206 | LP | field survey |
| -33.3292 | -70.2701 | 3246 | LP | field survey |
| -33.3289 | -70.2688 | 3299 | LP | field survey |
| -33.3249 | -70.2660 | 3348 | LP | field survey |
| -33.3235 | -70.2641 | 3405 | LP | field survey |
| -33.3246 | -70.2640 | 3430 | LP | field survey |
| -33.3254 | -70.2623 | 3467 | LP | field survey |
| -33.3607 | -70.2972 | 2345 | VN | field survey |
| -33.3703 | -70.2819 | 2389 | VN | field survey |
| -33.3704 | -70.2748 | 2445 | VN | field survey |
| -33.3664 | -70.2695 | 2498 | VN | field survey |
| -33.3636 | -70.2653 | 2549 | VN | field survey |
| -33.3618 | -70.2624 | 2599 | VN | field survey |
| -33.3622 | -70.2570 | 2648 | VN | field survey |
| -33.3611 | -70.2577 | 2704 | VN | field survey |
| -33.3605 | -70.2573 | 2741 | VN | field survey |
| -33.3577 | -70.2582 | 2790 | VN | field survey |
| -33.3582 | -70.2542 | 2855 | VN | field survey |
| -33.3590 | -70.2537 | 2897 | VN | field survey |
| -33.3573 | -70.2512 | 2953 | VN | field survey |
| -33.3565 | -70.2500 | 2994 | VN | field survey |
| -33.3516 | -70.2487 | 3059 | VN | field survey |
| -33.3491 | -70.2476 | 3080 | VN | field survey |
| -33.3469 | -70.2473 | 3153 | VN | field survey |
| -33.3437 | -70.2478 | 3195 | VN | field survey |
| -33.3332 | 70.2463 | 3256 | VN | field survey |
| -33.3401 | -70.2480 | 3284 | VN | field survey |
| -33.3320 | -70.2491 | 3350 | VN | field survey |
| -33.3297 | -70.2508 | 3382 | VN | field survey |
| -33.3267 | -70.2558 | 3444 | VN | field survey |
| -33.3256 | -70.2563 | 3473 | VN | field survey |

| | | | | |
|---|---|---|---|---|
| -70.3318 | -33.3488 | 1900 | LP | MIREN |
| -70.3277 | -33.3546 | 1998 | LP | MIREN |
| -70.3277 | -33.3578 | 2190 | LP | MIREN |
| -70.3201 | -33.3553 | 2265 | LP | MIREN |
| -70.2964 | -33.3426 | 2589 | LP | MIREN |
| -70.2917 | -33.3401 | 2674 | LP | MIREN |
| -70.2861 | -33.3303 | 2890 | LP | MIREN |
| -70.2803 | -33.3281 | 3061 | LP | MIREN |
| -70.2690 | -33.3292 | 3283 | LP | MIREN |
| -70.2657 | -33.3265 | 3370 | LP | MIREN |
| -70.2573 | -33.3251 | 3471 | LP | MIREN |
| -70.2586 | -33.3200 | 3585 | LP | MIREN |
| -70.2726 | -33.3679 | 2470 | VN | MIREN |
| -70.2645 | -33.3635 | 2554 | VN | MIREN |
| -70.2555 | -33.3631 | 2653 | VN | MIREN |
| -70.2578 | -33.3571 | 2784 | VN | MIREN |
| -70.2536 | -33.3599 | 2885 | VN | MIREN |
| -70.2477 | -33.3493 | 3070 | VN | MIREN |
| -70.2510 | -33.3418 | 3181 | VN | MIREN |
| -70.2506 | -33.3296 | 3377 | VN | MIREN |
| -70.2554 | -33.3259 | 3451 | VN | MIREN |

Table S4: Ecological traits and factors of 38 exotic species in study region. Residence time (First year report), Dispersal mode (A=Animal, W=Wind and Un=Unassisted) and life span (A=Annual; B=Biannual; P=Perennial). Data obtained from Fuentes et al. 2012).

| Specie | Family | Life span | Dispersal mode | Residence time |
|---|---|---|---|---|
| *Anagallis arvensis* | Primulaceae | A | Un | 1849 |
| *Anthemis cotula* | Asteraceae | A | Un | 1848 |
| *Avena barbata* | Poaceae | A | A | 1854 |
| *Bromus diandrus* | Poaceae | A | A | - |
| *Bromus scoparius* | Poaceae | A | A | 1875 |
| *Carduus nutans* | Asteraceae | B | W | 1960 |
| *Centaurea melitensis* | Asteraceae | A | A | 1848 |
| *Cerastium arvense* | Caryophyllaceae | P | Un | 1846 |
| *Chamomilla suaveolens* | Asteraceae | A | Un | - |
| *Chenopodium album* | Amaranthaceae | A | Un | 1948 |
| *Conium maculatum* | Apiaceae | B | Un | 1848 |
| *Convolvulus arvensis* | Convolvulaceae | P | Un | 1848 |
| *Cynoglossum creticum* | Boraginaceae | B | A | 1907 |
| *Echium vulgare* | Boraginaceae | A | Un | 1907 |
| *Erodium cicutarium* | Geraniaceae | A | Un | 1846 |

| | | | | |
|---|---|---|---|---|
| *Eschscholzia californica* | Papaveraceae | A | Un | 1881 |
| *Galium aparine* | Rubiaceae | A | A | 1848 |
| *Hirschfeldia incana* | Brassicaceae | A | Un | 1963 |
| *Hordeum murinum* | Poaceae | A | A | - |
| *Lactuca serriola* | Asteraceae | A | W | 1905 |
| *Lactuca virosa* | Asteraceae | A | W | 1933 |
| *Marrubium vulgare* | Lamiaceae | P | A | 1849 |
| *Onopordum acanthium* | Asteraceae | B | W | 1881 |
| *Plantago lanceolata* | Plantaginaceae | A | Un | 1910 |
| *Polygonum aviculare* | Polygonaceae | A | A | 1851 |
| *Rumex acetosella* | Polygonaceae | P | W | 1851 |
| *Rumex crispus* | Polygonaceae | P | Un | 1851 |
| *Sisymbrium orientale* | Brassicaceae | A | Un | 1929 |
| *Sonchus asper* | Asteraceae | A | W | 1848 |
| *Sonchus oleraceus* | Asteraceae | A | W | 1848 |
| *Tanacetum parthenium* | Asteraceae | P | Un | 1848 |
| *Taraxacum officinale* | Asteraceae | P | W | 1875 |
| *Tragopogon porrifolius* | Asteraceae | B | W | 1881 |
| *Trifolium glomeratum* | Fabaceae | A | Un | 1922 |
| *Tripleurospermum maritimum* | Asteraceae | B | Un | - |
| *Verbascum thapsus* | Scrophulariaceae | B | Un | 1881 |
| *Veronica persica* | Scrophulariaceae | A | A | 1909 |
| *Vulpia myuros* | Poaceae | A | W | - |
